# Cell-Autonomous and Non-Cell-Autonomous Antiviral Immunity via siRNA-Directed RNAi in *Drosophila melanogaster*

**DOI:** 10.70322/immune.2025.10001

**Published:** 2025-01-02

**Authors:** Haojiang Luan

**Affiliations:** Section on Neural Function, LMB, NIMH, National Institutes of Health, 35 Convent Drive, Bethesda, MD 20892, USA

**Keywords:** RNAi, siRNA, Antiviral immunity, Cell autonomous, Non-cell autonomous, Dcr-2, *Drosophila melanogaster*

## Abstract

In *Drosophila melanogaster*, the siRNA-directed RNAi pathway provides crucial antiviral defenses. Cell-autonomously, Dicer-2 (Dcr-2) recognizes and cleaves viral dsRNA into siRNAs, which are incorporated into the RNA-induced silencing complex (RISC). Argonaute 2 (Ago2) then targets and cleaves viral RNA, preventing replication. Non-cell-autonomously, infected hemocytes secrete exosomes containing viral siRNAs, spreading antiviral signals to other cells. Additionally, tunneling nanotubes can transfer RNAi components between neighboring cells, further enhancing systemic immunity. These findings highlight the sophisticated antiviral strategies in *Drosophila*, offering insights for broader antiviral research.

## 1. Introduction

Viral pathogens, including the recently emerged SARS-CoV-2 virus causing COVID-19 [1], are common causes of morbidity and mortality worldwide. Since viruses are obligate intracellular parasites, they must utilize the cellular pathways of infected host cells to replicate. Some viruses infect a limited number of hosts, while others have a broad host range. Arthropod-borne viruses (arboviruses) are one such group. Due to the difficulty of culturing many types of virus hosts in the laboratory, *Drosophila melanogaster* has been extensively used to study viral immunity and virus-host interaction [2].

*Drosophila melanogaster* has been a popular model for various biological studies, including genetics, development, neuroscience, disease modeling, and more [3–7]. The availability of genome sequences for many *Drosophila* species also makes it an excellent model for evolutionary studies. Moreover, many genetic tools and discoveries initially emerged in flies have been applied to other insects, worms, zebrafish, and mammalian systems, expanding the utility of this model [8–11].

In recent decades, *Drosophila melanogaster* has become a robust model for studying interactions between infectious pathogens (including bacteria, fungi, and viruses) and their hosts. This review highlights recent discoveries and advancements in small interfering RNA (siRNA)-directed RNAi viral immunity, focusing specifically on the mechanism of siRNA biogenesis, as well as the cell-autonomous and non-cell-autonomous antiviral defenses of the siRNA pathway in *Drosophila melanogaster*.

## 2. RNA Interference

RNA interference (RNAi), also known as Post-Transcriptional Gene Silencing (PTGS), is a conserved biological response to double-stranded (ds) RNA (dsRNA). It provides resistance to both internal parasitic and external pathogenic nucleic acids and silences cognate gene expression. RNAi was first described in the landmark work by Andrew Fire et al. [12]. In this work, they first identified dsRNA as the trigger of RNAi. Their finding in *C. elegans* was soon confirmed in other eukaryotes, such as plants, insects and mammals [13–15]. Since then, RNAi has become extensively studied and used as a tool for pest control and gene silencing in basic biological research. In 2006, Andrew Z. Fire and Craig C. Mello were awarded Nobel Prize in Physiology or Medicine for their work on RNAi.

The specificity of gene silencing by RNAi is determined by small silencing RNAs. In *Drosophila melanogaster*, there are at least three types of small silencing RNA: the siRNA, the microRNA (miRNA), and the Piwi-interacting RNA (piRNA). These small RNAs differ in their RNA precursor, RNA biogenesis processes, mechanism of action, associated effector proteins, target genes, and biological roles [16]. The core components of the RNAi pathway mediated by these small silencing RNA are Dicer [17] (Dcr) and Argonaute (Ago). In the fruit fly genome, there are two Dcr genes (Dcr-1 and Dcr-2) and five Ago genes (Ago1, Ago2, Ago3, Aub and Piwi). siRNAs and miRNAs bind to members of the Ago clade of Argonaute proteins, whereas piRNAs bind to members of the Piwi clade (more details below). Dicer is believed to have evolved through the fusion of distinct domains, including a helicase domain, an RNase III domain, and a PAZ domain, allowing it to process double-stranded RNA into siRNAs [18]. The diversification of the three RNAi pathways and their key components—Dcr and Ago—may result from adaptive evolution, aligning with their roles in regulation and defense [19–21]. However, a recent alternative neutral evolution hypothesis for this diversification has also been proposed [22].

### 2.1. The miRNA Pathway

**Figure 1.**
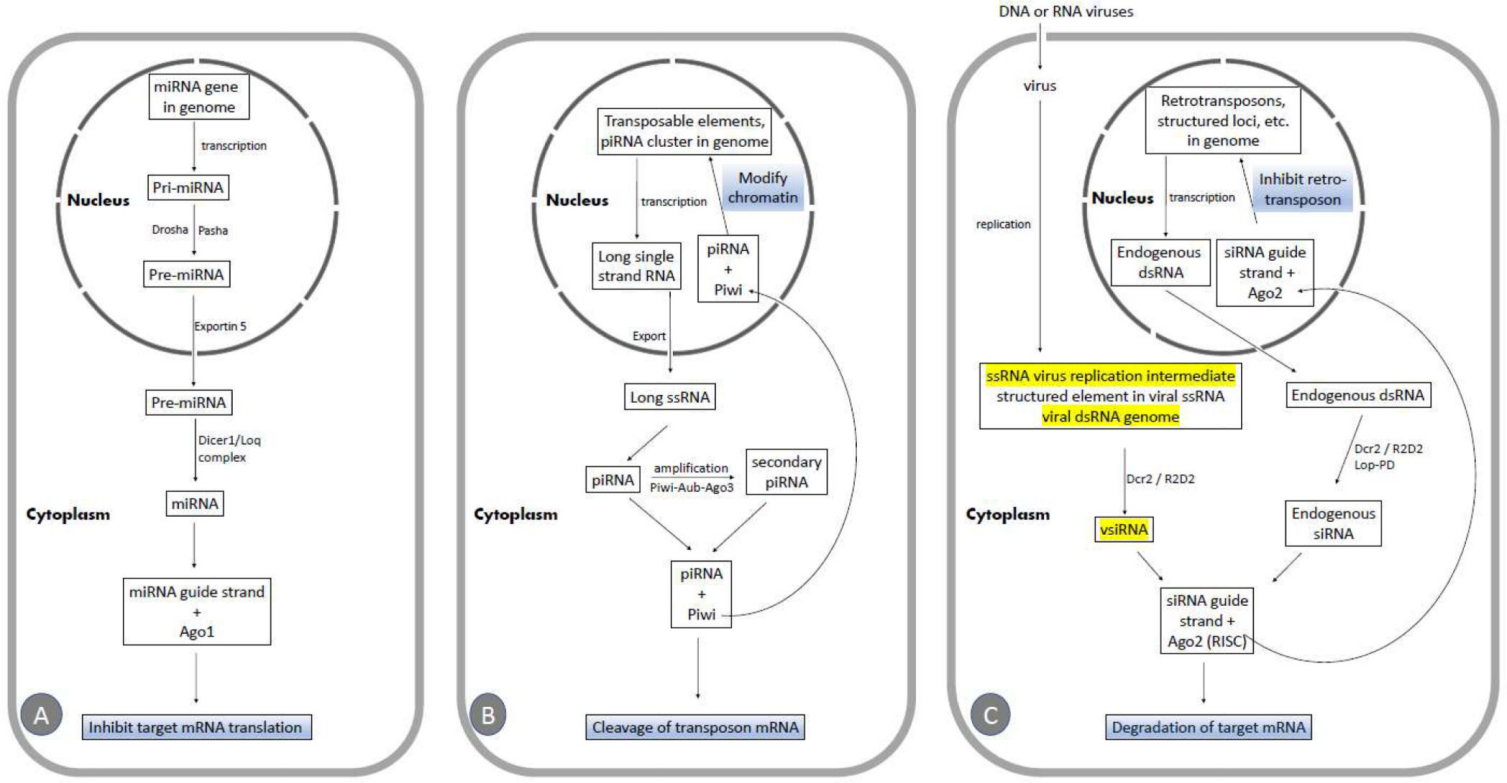
Schematic illustration of the three RNAi pathways. (**A**)The miRNA pathway. (**B**)The piRNA pathway. (**C**)The siRNA pathway. Targets of viral suppressors of RNAi (VSRs)are highlighted in yellow.

The miRNA pathway is crucial for animal development and cell differentiation [23,24]. miRNAs [25–30], which are a class of ubiquitously expressed RNAs of ∼22 nucleotides in length [31], and are encoded in the genome (Figure 1A), regulate the expression of potentially half of all coding genes in *Drosophila* [32,33]. miRNA genes are transcribed to produce primary miRNA (pri-miRNA) transcripts, which are then processed into pre-miRNA by the nuclear RNase III enzyme Drosha, along with the dsRNA binding protein Pasha [34]. pre-miRNAs are exported to the cytoplasm by Exportin5 [35,36] and processed into mature miRNAs by the Dicer1–Loquacious (Loqs) complex [28]. Alternatively, miRNAs can be located in introns (called mirtrons) and are released as authentic pre-miRNAs after splicing [36–38]. The guide strand is loaded into Ago1 [39], directing the miRNA to repress the translation of target RNAs (Figure 1A).

### 2.2. The piRNA Pathway

The piRNA pathway in fruit flies is primarily active in the animal germline [40–43] but may also be found in somatic tissues [44]. *Drosophila* piRNAs are small RNAs, 24–27 nucleotides in length, generated from single-stranded RNA (ssRNA) precursors [45,46], mostly derived from transposable elements and specific genome clusters (Figure 1B). Most piRNAs are antisense and are preferentially loaded into the proteins Piwi or Aubergine (Aub), while sense piRNAs are associated with Ago3. Piwi and AUB work together with Ago3 in an interdependent amplification cycle that produces additional piRNAs, maintaining the antisense bias of piRNA. Antisense piRNAs likely direct the cleavage of transposon mRNA or induce chromatin modifications at transposon loci (Figure 1B) [45,47]. It has been reported that the piRNA-directed RNAi pathway is not necessary for antiviral defense [48] in *Drosophila melanogaster*.

### 2.3. The siRNA Pathway and the Regulation of siRNA Biogenesis

#### 2.3.1. The Canonical siRNA Pathway

In *Drosophila melanogaster*, the canonical siRNA-directed RNAi pathway starts with the recognition of long dsRNA by Dcr-2, a dsRNA-specific RNase III family endonuclease. Dcr-2 associates with R2D2, a dsRNA-binding protein, and cleaves long dsRNAs to produce siRNA duplexes, which are 21 nucleotide long with 2 nucleotide overhangs [17,49,50]. These siRNAs are then loaded into the RNA-induced silencing complex (RISC) [51,52], in which the endoribonuclease Ago2 [53] cleaves the target RNA (Figure 1C). RISC is activated after the passenger siRNA strand [54,55] is removed by C3PO, a Mg2+-dependent endoribonuclease. In contrast, the guide strand remains associated with Ago2 in the RISC and is 2′-O-methylated on its 3′-terminal by the Hen1 methyltransferase [56], which stabilizes the silencing complex. The guide strand of the mature RISC base pairs with the complementary target mRNA, leading to Ago2-mediated cleavage of the target. AGO2 cuts its RNA target at the phosphodiester bond located between the nucleotides complementary to positions 10 and 11 of the guide strand [49,50].

In addition to exogenous dsRNA (such as viral dsRNA, transgenically expressed dsRNA, and dsRNA from injection or transfection), fly Dcr-2 also utilizes endogenous dsRNA (Figure 1C) as a precursor to produce endogenous siRNA, which represses retrotransposons in germline and somatic cells in *Drosophila*. This endogenous dsRNA originates from retrotransposons, 3′ regions of overlapping transcripts, long stem-loop structures from repetitive sequences located in intergenic regions on the X chromosome, and structured loci capable of forming long hairpin dsRNA [57–61].

#### 2.3.2. Dicing of dsRNA by Dcr-2

Dcr-2 has a conserved architecture comprising a helicase domain that hydrolyzes ATP [62], a domain of unknown function, two RNase III domains that mediate RNA cleavage, a platform domain, a Piwi Argonaute Zwille (PAZ) domain, and a dsRNA-binding motif [63]. DCR-2 recombinant proteins from *Drosophila* cells alone efficiently cleaved dsRNA into siRNA in an adenosine triphosphate (ATP)– and dose-dependent manner. It has been proposed that the helicase domain of Dcr-2 recognizes a long dsRNA substrate and then undergoes a conformational change [64]. The 5′-monophosphate of dsRNA is anchored by the phosphate-binding pocket in the Dcr-2 PAZ domain. The distance between this pocket and the RNA cleavage site in the RNase III domain determines the length of the produced siRNA [65].

*In vitro* biochemical and structural studies of Dcr-2 indicate that blunt dsRNA binds to the helicase domain, is locally unwound, and then threaded through the helicase domain in an ATP-dependent manner. This processive reaction produces multiple siRNAs from a single dsRNA before Dcr-2 dissociates. In contrast, dsRNAs with 3′ overhanging termini are cleaved in a distributive, ATP-independent manner, with Dcr-2 dissociating after each cleavage [66,67].

#### 2.3.3. How R2D2 and Loqs-PD Regulate dsRNA Dicing by Dcr-2

Some RISC-associated proteins regulate the siRNA-directed RNAi pathway. For example, Loqs isoforms PD and R2D2 proteins contain tandem-repeat dsRNA-binding domains and are partners of Dcr-2 proteins. R2D2 acts with Dcr-2 to load the siRNAs into Ago2 in *Drosophila* [68]. Without R2D2, Dcr-2 is destabilized *in vivo* [68]. Dcr-2 and R2D2 form a stable complex, and either protein alone is unstable. The association of Dcr-2 with R2D2 does not affect the ability of Dcr-2 to recruit or cleave dsRNA. The Dcr-2/R2D2 mutant complex is as active in siRNA production as the wild-type complex. This evidence suggests that R2D2 is not required for siRNA generation; instead, R2D2 might be necessary for stabilizing Dcr-2 in the siRNA-producing pathway [68]. In the Dcr-2-R2D2 complex, R2D2 detects the thermodynamic asymmetry of the siRNA and assists in loading the siRNA into Ago2 in a specific orientation. This process determines which strand of the siRNA duplex will be used by Ago2 as the guide strand [64]. *In vivo*, Loqs-PD isoforms, Dcr-2, and R2D2 may form a tertiary complex [69]. The Cryo-EM structure revealed that R2D2 and Loqs-PD can simultaneously bind to different regions of Dcr-2 without mutual interference [70]. The Dicer-2-Loqs-PD complex process exogenous siRNA precursor hairpins with long stems to generate siRNA [69]. Cryo-EM structures of Dcr-2–Loqs-PD in multiple states reveal that, upon dsRNA binding to Dcr-2, the N-terminal helicase and domain of unknown function 283 undergo conformational changes, creating an ATP-binding pocket and a 5′-phosphate-binding pocket. Subsequent ATP-dependent conformational changes result in an active dicing state that accurately cleaves the dsRNA into a 21 bp siRNA duplex [71].

## 3. The siRNA Directed Antiviral Immunity in *Drosophila melanogaster*

The first evidence of the small RNA directed RNAi pathway defense against viral infection came from research in plants. Twenty-five nucleotide RNAs complementary to the positive (genomic) strand of potato virus X (PVX) were detected four days after PVX inoculation in *Nicotiana benthamiana*. The presence of these 25-nucleotide RNAs is correlated with post-transcriptional gene silencing (PTGS) in the inoculated plant. We now have accumulated evidence showing that RNAi is the main antiviral defense in plants [72], nematodes [73], and insects [74,75], and recent findings suggest that RNAi may also play an antiviral role in mammals [76,77]. In *Drosophila melanogaster*, although a few other innate immunity pathways [78–80] (Toll, Imd, JAK-STAT) play antiviral roles, the RNAi pathway, especially the one directed by siRNA, is the most robust [81,82] antiviral pathway.

### 3.1. The Cell Autonomous Antiviral Immunity of siRNA-Directed RNAi

#### 3.1.1. All Major Types of Viruses Induce siRNA Directed RNAi to Restrict Virus Replication

The siRNA antiviral defense in *Drosophila*, induced by all major types of viruses—including (+) single strand(ss) RNA viruses, (−)ssRNA viruses, dsRNA viruses, and dsDNA viruses—have been well documented and reviewed in the literature [75,83–85]. A tabulated list of this information can be found in the review by Gammon DB and Mello CC [83]. The evidence generally falls into several categories:

- Cells or flies infected by viruses show initial virus replication and morbidity/mortality.
- Infection induces characteristic 21 nucleotide siRNA production.
- siRNA-induced RNAi can suppress viral replication.
- Mutation or depletion of RNAi pathway components, including Dcr-2 and Ago2, increases susceptibility to viral infection and more severe host morbidity/mortality.
- Many viruses encode viral suppressors of RNAi (VSRs) [86] to evade replication suppression by RNAi.

#### 3.1.2. Biogenesis of Viral siRNAs by Dcr-2

As in the canonical siRNA pathway, Dcr-2 is the core initiating component that senses and cuts viral long dsRNA in *Drosophila melanogaster*. Recent evidence shows that viral infection causes a rapid increase in Dcr-2 protein levels. Surprisingly, this increase does not correspond with a rise in Dcr-2 mRNA levels. The mechanism behind the induction of Dcr-2 proteins resembles translation on demand, suggesting that the siRNA pathway can be readily mobilized to fight against viral invasion [87].

Depending on the viral genome, the source of viral dsRNA precursors processed by Dcr-2 may include dsRNA viral genomes, replication intermediates of ssRNA viruses, structured elements in viral ssRNA (genomes or transcripts), and overlapping viral transcripts that hybridize to form dsRNA [83,88,89].

#### 3.1.3. The Biogenesis of vsiRNAs May Involve Distinct Mechanisms Compared to siRNAs Generated from Non-Viral Sources

Evidence suggests that the mechanism of siRNA biogenesis from viral dsRNA precursors, though extrinsic to cells, may differ from that of endogenous dsRNAs [90]. For example, an F225G point mutation in the Dcr-2 Walker A motif of the Hel2 subdomain reduced the level of endogenous siRNAs but did not significantly affect virus-derived siRNAs (vsiRNAs) [91]. The reason for this difference in mechanism is largely unknown. It could be attributed to the intrinsic features of viral dsRNA, expression of VSRs, virion packaging, or the effect of other co-factors of Dcr-2.

For instance, Loqs-PD is involved in the biogenesis of endogenous siRNA. However, mutations in Loqs alone or together with R2D2 exhibit no detectable negative impact on antiviral immunity against Flock house virus (FHV) virus in adult flies [92]. In another report, three days after challenging adult flies with Sindbis virus (SINV) and vesicular stomatitis virus (VSV), the viral genome levels of both SINV and VSV were much higher in R2D2 and Dcr-2 mutants than in wild-type flies. In contrast, Loqs mutants showed viral genome levels indistinguishable from wild-type. Host survival rates after virus challenge also correlated with changes in viral genome levels. These results suggest that Loqs-PD is dispensable for inhibiting virus replication and promoting host survival after infection [90].

Additionally, Arsenic resistance protein 2 (Ars2) interacts with Dcr-2 in *Drosophila* cells [93]. Silencing of Ars2 led to increased replication of VSV, Drosophila C virus (DCV), FHV, and SINV. However, the antiviral function of Ars2 seems to be specific to RNA viruses, as depletion of Ars2 did not affect infection with the dsDNA vaccinia virus.

#### 3.1.4. vsiRNA Profiles Resulting from Various Viral Infections

Most vsiRNAs are 21 nucleotides in length, which is the expected size of Dcr-2 cleavage products. Depletion of Dcr-2 nearly abolished all vsiRNA biogenesis [88]. Next-generation sequencing revealed that viral dsRNAs generate reproducible spectra of siRNAs through the *Drosophila* RNA silencing machinery. A few interesting features of vsiRNAs are worth highlighting.

- Different viruses exhibit unique patterns of vsiRNA production: For example, Rift Valley fever virus (RVFV), a (−) single-strand RNA virus, produces vsiRNAs not from dsRNA replication intermediates but from a structured viral RNA hairpin in a discrete intergenic region, S-segment. VSV, a (−) ss RNA virus, and RVFV vsiRNAs are derived from both genomic and antigenomic RNA strands in roughly equal ratios [88], whereas the majority of the vsiRNAs produced during infection of *Drosophila* cells with DCV and West Nile Virus [94], both of which are (+) ssRNA viruses, map to the genomic strand.
- DNA Viruses: DNA viruses can also elicit vsiRNA production and may have evolved strategies to harness or counteract RNA silencing machinery [92,95,96]. For example, vsiRNAs produced during vaccinia virus infection were identified. These vsiRNAs are particularly derived from structured hairpins encoded by terminal repeat sequences [92].

#### 3.1.5. Polyadenylated Viral RNA Is the Preferred Target of vsiRNA-Directed RNAi

Multiple lines of evidence suggest that viral polyadenylated RNA is the preferred target of vsiRNAs [16,90,97,98] to inhibit viral replication. Ago2, the core component of RISC that slices target RNA, is associated with ribosomes. It has been proposed recently that Ago2 scans viral mRNAs before they are translated by the cellular machinery. When the guide strand of vsiRNA hybridizes with the cognate viral mRNA, Ago2 cuts the viral mRNA, preventing its translation and thereby inhibiting viral replication [98].

#### 3.1.6. Viral DNA (vDNA) from Viral RNA: An Alternative Pathway for siRNA Biogenesis

In plants and nematodes, effective antiviral RNAi involves the amplification of siRNAs by host RNA-dependent RNA polymerases (RdRPs) following the produce of the primary siRNA from viral dsRNA replicative intermediates [99]. However, fruit flies lack RdRP homologs yet still exhibit abundant vsiRNA, similar to plants and nematodes. This suggests that there may be an alternative pathway for the amplification of primary viral siRNAs in fruit flies. Poirier et al. [100]. recently demonstrated the presence of circular viral DNA in fruit fly S2 cells and adults infected with Flock House Virus (FHV), a bipartite positive-strand RNA virus. In this pathway, viral RNA is reverse transcribed into viral DNA (vDNA) by cellular reverse transcriptase [101]. They showed that FHV-derived extrachromosomal circular DNA (eccDNA) molecules accumulate in both *in vitro* and *in vivo* settings. Deep sequencing of these eccDNA molecules confirmed they comprised a heterogeneous population of chimeric, partial, and truncated viral genomic sequences, similar to previously characterized [101]. In addition, the authors showed that the viral eccDNA serves as a template for viral dsRNA production, revealing a novel biogenesis pathway for vsiRNAs [100]. Inhibition of reverse transcription with AZT increases host susceptibility to infection and reduces vsiRNA biogenesis, indicating that viral eccDNA production is integral to the insect antiviral RNAi response. Injection of eccDNA from FHV-infected cells into naive flies induced vsiRNA production and modestly increased fly survival against subsequent FHV infection. The induced siRNAs were primarily 21 nucleotides in length with 5′ monophosphate ends, mapping uniformly across the viral genome. This distribution pattern differed from the vsiRNAs in FHV-infected flies, which are mostly positive-strand and target the 5′-terminal regions of the viral genomic RNAs. This difference suggests that eccDNA-derived vsiRNAs and vsiRNAs in FHV-infected flies represent two distinct populations of siRNAs. The moderate protection against subsequent FHV infection in fruit flies is likely because effective antiviral RNAi requires viral siRNAs derived from dsRNA precursors generated during both viral RNA replication and RNA transcription that uses the viral eccDNAs as templates. Viral eccDNA production is a conserved feature in insects, including mosquitoes infected with the chikungunya virus (CHIKV). SINV and CHIKV infections in mosquitoes produced both linear and circular viral DNA forms [100]. The findings on viral eccDNA in fruit flies and mosquitoes open new avenues for studying vsiRNA biogenesis and function.

Mondotte et al. demonstrated [102] that antiviral transgenerational immune priming occurs in Drosophila and mosquitoes following parental exposure to different single-stranded RNA viruses. This protection is virus-specific, targeting the same virus, but it is RNAi-independent and persists for several generations [102]. vDNA is thought to play a key role in this antiviral immune memory, as it has been detected in adult flies infected during the larval stage [103] and in the offspring of infected adult females [102]. However, the mechanisms underlying the transgenerational transfer and amplification of vDNA remain unclear.

#### 3.1.7. Mechanisms Developed by Viruses to Evade siRNA-Mediated Antiviral Immunity

Given the critical role of RNAi in restricting diverse viral infections, it is unsurprising that many viruses have evolved multiple strategies to evade RNAi responses, including VSRs [86] and RNA decoys.

- VSRs: DCV encodes an VSR, DCV-1A, which binds dsRNA (Figure 1C), protecting the viral dsRNA replication intermediate during infection. This explains the skewed vsiRNA distribution pattern, as the antiviral machinery is forced to target other viral RNA species. This may be a common mechanism utilized by different kinds of viruses [104]. For example, FHV also carries an RNAi suppressor, B2, that binds siRNAs and long dsRNAs (Figure 1C). vsiRNAs generated during wild-type virus infection are also skewed toward the genomic (+) strand, whereas vsiRNAs map to both (+) and (−) strand genomes of FHV when flies are infected by a virus strain that does not express B2, FHVΔB2 [92].
- RNA Decoys: Some viruses may employ RNA decoys to evade the RNAi machinery. For example, VSV defective interfering (DI) particles and the RVFV S segment hairpin may serve as decoys, diverting Dcr-2 activity away from essential viral RNAs, thereby allowing the virus to partially evade the antiviral siRNA pathway [92].

## 4. The Non-Cell Autonomous Antiviral Immunity through siRNA-Directed RNAi

The above cell-autonomous processes occur within the infected cell, providing protection only to that specific cell. It has generally been believed that fruit flies lack an adaptive immune system and rely solely on innate immunity to protect against pathogens. However, emerging evidence has challenged this notion. Infected cells in *Drosophila* can also convey non-cell-autonomous antiviral immunity through the spread and uptake of viral RNA.

### 4.1. Hemocyte-Exosome Pathway

Exosomes are a class of extracellular vesicles that originate from the inward budding of the plasma membrane and can be secreted into body fluids such as saliva, blood plasma, breast milk, and urine. Exosomes can act as vehicles for the long-range transport of biologically active molecules, including proteins, mRNA, and miRNA [81,105]. The transported mRNA can be translated into proteins, while the miRNAs target host mRNAs in recipient cells. Engineered exosomes have been used to deliver drugs, miRNA, and siRNA-based therapeutic molecules to specific organs [106,107]. Additionally, many viruses use exosomes to export their viral elements within cellular compartments [108]. These features make exosomes a great candidate for spreading RNAi pathway components and conveying systemic antiviral immunity to recipient cells.

Recently, Tassetto et al. [109] demonstrated that fruit flies have a systemic siRNA-mediated RNAi pathway, spread through exosomes released by circulating hemocytes. They demonstrated that haemocytes take up dsRNA from infected cells. Then, vDNA is synthesized in haemocytes through endogenous transposon reverse transcriptase and used as a template for the de novo synthesis of secondary siRNAs, which are then secreted in exosomes. More importantly, exosomes containing viral siRNAs, purified from the hemolymph of infected flies, provide passive protection against virus challenges in naive animals. Hemocytes isolated from flies after three weeks of infection still contained vDNAs, suggesting that vDNAs facilitate the amplification of antiviral responses and provide immunological memory for a prolonged defense. Similarly, it has been recently reported that siRNAs are found and secreted in extracellular vesicles in cultured red flour beetle (*Tribolium castaneum*) cells [110]. siRNA is amplified in hemocytes and then secreted; this pathway can protect all the cells that hemolymph can reach.

One of the remaining questions is the source of the dsRNA taken up by circulating hemocytes. A study by Chen YG and Hur S suggests that the dsRNA may come from virus-infected cells [111], but this possibility needs further verification. It has been shown that hemocytes engulf virus-infected cells through phagocytosis [112,113]. A reasonable hypothesis is that the engulfed cells may release dsRNA, which serves as a template for viral complementary DNA synthesis.

### 4.2. Tunneling Nanotube Pathway

Another potential non-cell autonomous RNAi antiviral mechanism has been reported in cultured fruit fly cell lines. Tunneling nanotubes are cytoskeletal protrusions that extend from the plasma membrane and connect cells over long distances, allowing the intercellular transfer of large cargo, including proteins and RNA [114]. It has been shown that the fruit fly testis has microtubule-based nanotubes resembling tunneling nanotubes in mammalian cells [115]. Neighboring cultured *Drosophila* S2 cells are connected by similar nanotube-like structures, which can be induced by infection with either FHV or DCV, but not by bacterial infection. Following FHV infection, Ago2 and dsRNA were observed along these nanotube-like structures. Additionally, Ago2 was found inside the tubules and could be transferred from infected cells to uninfected cells. This evidence suggests that the nanotube-like structures in S2 cells could be a mechanism by which antiviral RNAi machinery spreads from infected cells to uninfected cells, triggering antiviral immunity [116]. Compared to the hemocyte-exosome pathway, viral protection via tunneling nanotubes can only reach nearby cells.

In line with this, human AGO2 resides in nanotube structures along with other components of the RNAi machinery (including Drosha, DGCR8, and Dicer) in multiple cultured human cell lines [117]. However, this mechanism still needs to be verified by *in vivo* studies.

### 4.3. Conclusions

In addition to providing cell-autonomous antiviral protection, the siRNA pathway in *Drosophila* engages in systemic immune responses through non-cell-autonomous mechanisms, such as exosome signaling and nanotube structures. This dual-mode immunity reflects the complexity of *Drosophila*’s response to viral infection and its potential applications as a model for studying antiviral defense mechanisms in other organisms. Confirming these findings across different models will significantly enhance our understanding of RNAi-based immunity, benefiting both evolutionary and applied research.

## 5. Closing Remarks

It is important to note that most, if not all, of the above general observations are not universally applicable to all siRNA-directed RNAi pathways. Many components and mechanisms of the RNAi pathway observed in fruit flies are well conserved across other insects. Therefore, it is crucial to validate findings in *Drosophila* using other model organisms, and vice versa, as this cross-validation will significantly enhance our understanding of the RNAi pathway.

The most common route of viral entry in nature is likely oral infection. However, most studies involving *Drosophila melanogaster* and viruses use injection for infection. Additionally, *Drosophila* is not the natural host for some of the viruses studied. It has been reported that the same pathogen can elicit different immune responses within the same organism depending on the route of delivery [103,118]. Therefore, it is important to consider whether the observed responses accurately reflect what occurs in natural hosts and during natural infections.
